# Heated Tobacco Products: Current Evidence of Pulmonary Harm and Lung Cancer Risk

**DOI:** 10.7759/cureus.106926

**Published:** 2026-04-12

**Authors:** Beatriz L Albuquerque, Inês M Coelho, Inês L Albuquerque, Helder Lanhas, Ana F Vilaça

**Affiliations:** 1 Family Medicine, Unidade de Saúde Familiar (USF) Manuel Rocha Peixoto, Braga, PRT; 2 Family Medicine, Unidade de Saúde Familiar (USF) Saúde Oeste, Braga, PRT; 3 Family Medicine, Unidade de Saúde Familiar (USF) Martim, Braga, PRT

**Keywords:** biomarkers of exposure, biomarkers of potential harm, conventional cigarettes, heated tobacco products, lung cancer, pulmonary harm, risk perception, smoking cessation

## Abstract

Heated tobacco products (HTPs) are promoted as less harmful alternatives to conventional cigarettes (CCs) because they avoid combustion and reduce the formation of toxic compounds. However, evidence supporting a real reduction in lung cancer (LC) risk remains limited and is largely based on biomarker studies rather than long-term epidemiological data. This review assessed the association between HTP use and LC risk, examined the impact of reduced-exposure claims on consumer perceptions, and evaluated the role of HTPs in smoking cessation. A literature search of PubMed, Medline, and the Cochrane Library identified 26 studies published between January 2020 and June 2025. Available evidence indicates that HTPs generate substantially lower levels of several carcinogenic compounds and are associated with reductions in biomarkers of exposure and biomarkers of potential harm compared with CCs. Risk estimation models suggest that the excess LC risk associated with HTP use may represent approximately 3-4% of that observed among CC smokers; however, these projections rely on indirect measures and carry considerable uncertainty. Although HTPs may contribute to reduced toxic exposure, they are not risk-free and continue to induce biological alterations linked to inflammation and oxidative stress. Reduced-exposure claims influence consumer risk perceptions and may encourage product uptake. In the context of smoking cessation, HTPs may reduce CC consumption but frequently result in dual use rather than complete abstinence. Independent longitudinal studies are required to clarify their long-term impact on LC incidence and public health.

## Introduction and background

Lung cancer (LC) is the most common type of cancer and represents the leading cause of cancer-related mortality worldwide. According to global estimates, LC caused about 2.09 million new cases in 2018 (11.6% of all cancers) and 1.76 million deaths, accounting for 18.4% of all cancer-related deaths. It is particularly prevalent among tobacco users, with approximately 71% of LC cases associated with active tobacco smoking and 1% attributed to passive exposure [1]. Smoking remains the leading single preventable cause of death, accounting for more than eight million deaths annually, including approximately 1.2 million non-smokers involuntarily exposed to tobacco smoke. It is estimated that there are currently around 1.3 billion smokers worldwide, representing 22.3% of the global population [2-4], despite increasing awareness of the risks associated with tobacco use.

In light of the burden of the tobacco epidemic, the tobacco industry has sought to develop and promote alternatives to conventional cigarettes (CCs), purportedly less harmful. Among these, heated tobacco products (HTPs) stand out; they were introduced to the market over the past decade and have rapidly spread across several countries [3,5]. IQOS®, developed by Philip Morris International and launched in Japan in 2014, has become the most emblematic case, expanding to dozens of markets and, in some countries, achieving significant shares of total tobacco sales [6].

The U.S. Food and Drug Administration (FDA) authorized the commercialization of HTPs in the United States in April 2019. Subsequently, in July 2020, it granted a “Modified Risk Tobacco Product” (MRTP) order, authorizing the use of the following reduced-exposure claim: “the IQOS system heats tobacco but does not burn it; this significantly reduces the production of harmful and potentially harmful chemicals; scientific studies have demonstrated that completely switching from conventional cigarettes to the IQOS system significantly reduces your body’s exposure to harmful or potentially harmful chemicals”. However, IQOS was removed from all U.S. markets in November 2021 as a result of a “Cease-and-Desist Order” (CDO) issued by the U.S. International Trade Commission (ITC) in September of the same year [7]. The reason was not health-related (i.e., related to toxicity), but rather legal, stemming from a patent dispute with British American Tobacco, which led the ITC to prohibit the importation of the product [8].

HTPs differ from CCs in that they use electronic devices to heat processed tobacco to controlled temperatures (240-350 °C) without reaching combustion. Unlike CCs, which contain natural tobacco leaves (TL), HTPs sticks typically use reconstituted tobacco leaves (RTL), processed tobacco material formed from ground tobacco, additives, and humectants, designed to enable controlled heating and aerosol generation without ignition. This process generates aerosols containing nicotine and other chemical compounds, generally at lower concentrations than those observed in cigarette combustion [2,9]. The reduction in emissions of harmful and potentially harmful constituents has been estimated by the tobacco industry itself to be as high as 90-95% compared with CC smoke [7]. However, an average reduction in the concentrations of these constituents does not necessarily imply a corresponding reduction in risk for consumers who switch to these products [10]​. Toxic compounds relevant to carcinogenesis, such as nitrosamines, polycyclic aromatic hydrocarbons, and carbonyl compounds, are not completely eliminated [9,11]. In addition, HTPs contain additional toxic compounds, such as formaldehyde and elevated concentrations of acenaphthene, both of which have potentially carcinogenic properties [4].

CC smoke contains approximately 7,000 chemical compounds, including 79 carcinogens. The formation of these compounds is closely linked to complex thermochemical processes, including combustion and pyrolysis [2,3,12]. Given that the health risks associated with smoking are primarily attributed to harmful constituents generated by combustion, it has been proposed that smokers may benefit from switching to HTPs [7]. 

Despite the promise of harm reduction, HTPs face important limitations in the available scientific evidence. The main challenge is the latency between tobacco exposure and the development of LC, which may extend over decades. Consequently, robust epidemiological data on LC risk associated with exclusive HTPs use are unlikely to be available in the short term [13]. To date, most clinical evidence derives from short-term trials focused on biomarkers of exposure (BoE) and biomarkers of potential harm (BoPH), which suggest intermediate risk reductions between smoking cessation and continued use of CCs [14-22].

Another central issue is the influence of marketing and "reduced exposure" claims on consumer perceptions. Advertising for HTPs consistently emphasizes reductions of more than 90% in toxic substances, which many smokers interpret as equivalent to reduced risk [6]​​​​​​​. Qualitative and quantitative studies show that smokers and former smokers perceive IQOS as less harmful than CCs, although uncertainties regarding its actual safety remain [8]​​​​​​​. This association contributes to product adoption, particularly among adolescents and young adults, who are often sensitive to marketing strategies linked to innovation and aesthetics [23,24]​​​​​​​.

Physicians are often regarded as role models for health-related behaviors and play a key role in smoking cessation practices. Owing to the exponential growth in HTPs use, together with aggressive tobacco industry marketing, physicians have increasingly engaged with smoking patients regarding the use of HTPs. However, many physicians appear to be insufficiently prepared to provide informed and consistent counseling, with substantial variability in attitudes observed according to age, medical specialty, and personal smoking habits. Therefore, evidence-based guidelines are crucial to support physicians in counseling patients about HTPs [5].​​​​​​​

In parallel, the role of HTPs in smoking cessation or substitution has been debated. Although complete smoking cessation is the most effective strategy to reduce the risk of tobacco-related diseases, many smokers are unable to quit [14-16]​​​​​​​. In this context, HTPs are presented as harm reduction tools, with the substitution of CCs by HTPs being proposed. Observational studies indicate that, in some settings, exclusive HTP use has been associated with a reduction or cessation of CC consumption [7]​​​​​​​. However, other studies indicate that dual use (CCs plus HTPs) is common, potentially perpetuating nicotine dependence and undermining the potential benefits [25,26]​​​​​​​.

Thus, although HTPs represent a relevant innovation in the tobacco product landscape, it remains unclear whether their use effectively translates into a strategy for reducing pulmonary harm or instead primarily reflects a commercial repositioning of the tobacco industry, supported by claims of lower exposure to harmful compounds, but still underpinned by limited and inconclusive epidemiological evidence.

In this context, the present evidence-based narrative review seeks to critically examine the current scientific evidence regarding HTPs in comparison with CCs. Specifically, it aims to assess whether the use of HTPs is associated with a reduced risk of LC relative to CCs, to explore the relationship between industry claims of reduced exposure and consumer perceptions and intentions to use these products, and to examine the extent to which HTPs function as tools for smoking substitution or cessation. By integrating epidemiological, toxicological, and behavioral evidence, this review intends to provide a balanced and well-grounded evaluation of the actual role of HTPs in pulmonary harm reduction.

## Review

Methods

This review was conducted as an evidence-based narrative review and was not designed as a formal systematic review. Therefore, PRISMA guidelines and protocol registration were not applied.

To formulate the research question, this evidence-based narrative review was guided by the PICO framework, comprising the following elements: target population - adult smokers (≥18 years); intervention/exposure - use of HTPs; comparator/control - use of CCs; outcome - pulmonary harm and LC risk, assessed through BoE, BoPH, and risk estimation models. Secondary outcomes included consumer risk perception and smoking behavior in response to reduced-exposure claims. Based on this framework, the research question was defined as: “What is the current evidence on pulmonary harm and LC risk associated with the use of HTPs compared with CCs?”

A literature search was conducted to identify studies relevant to the research question. The databases consulted were PubMed, Medline, and the Cochrane Library. Publications in Portuguese and English published between January 2020 and June 2025 were included. The search string consisted exclusively of the Medical Subject Headings (MeSH) term “Heated Tobacco” in each database (heated tobacco[MeSH Terms]). No Boolean operators or additional free-text terms were applied. Given the relatively recent introduction of the MeSH term “Heated Tobacco” and the evolving indexing of this topic, the search strategy prioritized sensitivity. Exploratory searches showed that adding additional terms reduced retrieval of relevant records; therefore, a single MeSH term was used. It is also noteworthy that all types of publications and studies were included, regardless of methodological design, in order to ensure that no potentially relevant information was excluded from this review.

The initial search retrieved 540 publications, distributed as follows: PubMed/Medline (n=434), and Cochrane Library (n=106). Records were assessed for eligibility according to predefined criteria.

Studies were included if they met the following criteria: (i) comparative investigations between heated tobacco use and conventional tobacco use; (ii) evaluation of outcomes related to lung neoplasia, including risk, biomarkers, and lesion progression; and (iii) both independent studies and studies sponsored by the tobacco industry. Exclusion criteria were: (i) experimental studies conducted in animal models; (ii) duplicate publications or studies not available in full text; and (iii) studies that did not meet the predefined inclusion criteria.

The study selection process was carried out in two sequential stages: (i) screening of titles and abstracts to identify potentially eligible publications and (ii) full-text review of the preselected articles. All stages were conducted independently by three reviewers, with any disagreements resolved by consensus. In the final analysis, 26 studies were included.

Data extraction was performed using a spreadsheet developed in Microsoft Excel, completed independently by the three reviewers. The collected information included year of publication, study title, objectives, introduction, methodology, main results, discussion, conclusions, and reported limitations.

The strength of recommendation was determined according to the original Strength of Recommendation Taxonomy (SORT) framework proposed by Ebell et al. [27] (see Appendices), based on two sequential steps: (1) classification of the level of evidence (Level 1-3) according to study design and methodological quality, and (2) attribution of a strength of recommendation (A-C) based on the consistency and quality of patient-oriented evidence across studies. Each included study was independently assessed by the three reviewers according to these criteria, and discrepancies were resolved by consensus.

Results

LC Risk in HTPs vs CCs: Risk Estimates

Table 1 summarizes the main studies evaluating LC risk estimates comparing HTPs and CCs.

**Table 1:** Summary of studies evaluating LC risk estimates comparing HTPs and CCs, with the corresponding SORT classification. CC: Conventional cigarette; HTP: Heated tobacco product; LC: Lung cancer; SORT: Strength of Recommendation Taxonomy

Study	Result	SORT
Lee et al. 2025 [2] (independent study)	The relative formation of carcinogenic compounds in CCs was more than twice that observed in HTPs.	Strength B, Level 2
Lee et al. 2025 [13] (industry-linked study: no direct funding declared)	The estimated relative risk for LC among HTP users was 1.44: 3.4% of the excess risk observed in CC smokers.	Strength B, Level 2
Espositoet al. 2022 [12] (independent study)	Exposure to acrylamide from HTPs is approximately fivefold lower than that observed with CCs, suggesting a lower carcinogenic risk.	Strength B, Level 2
Rodrigo et al. 2020 [10] (industry-sponsored study)	The relative risk of developing cancer associated with HTP use was 3.9% when compared with the risk associated with CCs consumption.	Strength B, Level 2

Lee et al*.* compared the toxic compounds formed during tobacco pyrolysis in CCs and HTPs under controlled experimental conditions that simulate their respective smoking mechanisms. Importantly, the analysis used natural TL (obtained from CCs) and RTL (processed tobacco material specifically manufactured for HTP sticks), reflecting the actual composition of each product type. The authors observed that, in CCs, the pyrolysis of tobacco leaves occurs over a wide temperature range (200-800°C), leading to a significant increase in the formation of toxic compounds, including substances associated with respiratory, cardiovascular, neurological, and carcinogenic effects. In contrast, in HTPs, controlled heating at approximately 350°C limited the thermal degradation of lignocellulosic components, resulting in a substantially lower production of carcinogens. When the formation of carcinogenic compounds was normalized to nicotine, used as an indicator of dependence, the relative exposure to carcinogens in CCs was found to be more than twice that observed in HTPs [2].

Lee et al. developed a quantitative framework to estimate the risk of LC associated with the use of HTPs in the absence of robust epidemiological evidence, by integrating BoE and BoPH. Through a systematic review of 38 studies conducted in North America and Europe, regression models were constructed to link biomarker concentrations to the relative risk (RR) of LC, using the established risks of CCs and other well-characterised tobacco products as reference comparators. Based on biomarkers demonstrating a statistically significant association with cancer risk (p < 0.01) and adequate model performance in non-user populations, HTP use was estimated to be associated with an RR of 1.44 (95% CI: 0.41-5.08). This corresponds to an excess risk of approximately 3.4% relative to that observed among CC smokers. Despite acknowledged methodological limitations and uncertainty reflected in the wide confidence intervals, the authors conclude that, according to the biomarker-based evidence, HTPs are associated with a substantially lower risk of LC than CCs [13].

Esposito et al. evaluated acrylamide levels, a compound recognized as carcinogenic, by comparing its concentration in the aerosol of CCs and HTPs. The authors observed that HTPs exhibited significantly lower acrylamide levels than CCs (99-187 ng/cigarette vs. 235-897 ng/cigarette, respectively), with median values approximately fivefold lower (p < 0.05). In the assessment of incremental lifetime cancer risk, consumption of 40 CCs per day exceeded the reference value established by the U.S. Environmental Protection Agency (>1 × 10⁻⁴), indicating a potential cancer occurrence in more than 1 in 10,000 individuals. By contrast, HTPs were associated with lower risk estimates, although in some scenarios these exceeded the more conservative threshold defined by Health Canada (1 × 10⁻⁵) at low to moderate consumption levels (<15 HTPs per day) [12].

Rodrigo et al. conducted a comparative assessment of the carcinogenic risk associated with the consumption of CCs and HTPs. The mean lifetime cancer risk values for CCs ranged from 1.40 × 10⁻² to 3.97 × 10⁻², with a median of 2.73 × 10⁻², whereas for HTPs the estimated range was substantially lower, spanning from 4.53 × 10⁻⁵ to 3.95 × 10⁻³, with a median of 1.06 × 10⁻³. This corresponds to an approximate 25-fold reduction in risk compared with CCs. Based on median values, the RR of developing cancer associated with HTP use was 3.9% of that associated with CC consumption. Despite this substantial reduction, the risk associated with HTPs remains above the acceptability threshold of 10⁻⁴ established by some health authorities, although it is markedly lower than that observed for CCs [10].

LC Risk in HTPs vs CCs: Biomarker Analysis

Table 2 summarizes studies evaluating BoE and BoPH associated with HTP use compared with CCs.

**Table 2:** Summary of studies evaluating BoE and BoPH associated with HTP use compared with CCs, with the corresponding SORT classification. HTP: Heated tobacco product; BoE: Biomarkers of exposure; BoPH: Biomarkers of potential harm; COHb: Carboxyhemoglobin; NNAL: 4-(methylnitrosamino)-1-(3-pyridyl)-1-butanol; WBC: White blood cell; 8-epi-PGF2α: 8 epi-prostaglandin-F2α; CC: Conventional cigarette; SORT: Strength of Recommendation Taxonomy.

Study	Result	SORT
Śniadach et al. 2025 [4] (independent study)	(1) Users of HTPs exhibited significantly lower levels of BoE and BoPH (including COHb, urinary NNAL, WBC count, 8-epi-PGF2α, among others) at both 1 and 2 years of use compared with CC smokers. (2) The biomarker panels employed showed changes in the same direction as those observed following smoking cessation; however, values remained higher than those observed in never-smokers.	Strength C, Level 3
Ansari et al. 2025 [14] (industry-sponsored study)		Strength B, Level 2
Ansari et al. 2024 [15] (industry-sponsored study)		Strength B, Level 1
*Gale et al.* 2022 [16] (industry-sponsored study)		Strength B, Level 1

Śniadach et al. conducted a comprehensive narrative review examining changes in BoE among users of CCs and HTPs, with the aim of evaluating differences in inflammatory, oxidative, and biological damage profiles associated with each mode of consumption. Consistently, CC use was associated with more pronounced increases in pro-inflammatory cytokines (including TNF-α, IL-6, IL-8, and IL-17), markers of systemic inflammation (C-reactive protein, CRP), tissue-degrading enzymes (matrix metalloproteinase-9, MMP-9), and indicators of oxidative stress, suggesting a state of chronic inflammation and greater potential for cellular damage. By comparison, HTPs generally exhibited a lower overall impact on the biomarkers analyzed, with changes that were limited and often transient, particularly in inflammatory parameters, although they were not devoid of adverse biological effects. The authors conclude that, while HTPs may represent a reduction in biological exposure to harmful compounds relative to CCs, the variability of findings and the scarcity of long-term data preclude definitive conclusions regarding their risk profile, underscoring the need for additional longitudinal and clinical studies [4].

Ansari et al*.* integrated evidence from a 12-month randomized controlled trial and a cross-sectional real-world study to evaluate the impact of replacing CCs use with a HTP on BoPH associated with cardiovascular, respiratory, and carcinogenic diseases. In the clinical trial, 984 adult smokers were randomized (1:1) either to continue smoking or to switch completely or predominantly to an HTP. Participants who switched demonstrated favorable and consistent improvements over 12 months across eight core BoPH - including carboxyhemoglobin (COHb), total 4-(methylnitrosamino)-1-(3-pyridyl)-1-butanol (NNAL), white blood cell (WBC) count, 8 epi-prostaglandin-F2α (8-epi-PGF2α), HDL cholesterol, soluble intercellular adhesion molecule-1 (sICAM-1), 11-dehydrothromboxane B2 (11-DTX-B2), and predicted forced expiratory volume in 1 second % (FEV1%), with trajectories comparable to those observed following smoking cessation and distinct from those recorded in participants who continued to smoke. However, the 12-month extension was exploratory and relied on unadjusted P-values without correction for multiplicity; participants were not required to abstain completely from cigarettes, allowing dual use, and product consumption was largely self-reported, which may not fully reflect actual exposure. Moreover, the study population consisted of generally healthy smokers and assessed surrogate biomarkers rather than clinical outcomes. These findings were corroborated by the cross-sectional study, in which HTP users for ≥2 years displayed significantly more favorable levels across all nine BoPH assessed compared with current smokers, with overall values approaching those observed in former smokers. Taken together, the two studies suggest that sustained substitution of CCs with HTPs is associated with reduced biological exposure and improvements across multiple early smoking-related pathophysiological pathways; however, the authors emphasize that these biomarkers represent indirect indicators of risk and do not replace the need for long-term clinical and epidemiological data [14,15].

Gale et al. assessed, in a 12-month ambulatory clinical study, changes in BoE and BoPH among smokers who continued to smoke CCs, those who switched exclusively to an HTP, those who achieved complete tobacco cessation, and never-smokers. After 360 days, participants who replaced CC use with HTPs exhibited substantial and sustained reductions in most BoE associated with smoke toxicants, including acrylonitrile, benzene, 1,3-butadiene, acrolein, and carbon monoxide (CO), with magnitudes generally comparable to those observed in the cessation group, although reductions were less pronounced for tobacco-specific nitrosamines (NNAL and N-nitrosonornicotine (NNN)) and for nicotine exposure. In parallel, several BoPH related to inflammation, oxidative stress, endothelial function, and cardiovascular parameters (such as 8-epi-PGF2α, WBC, sICAM-1, HDL cholesterol, and fractional exhaled nitric oxide (FeNO)) evolved in a favorable direction in both the HTP and cessation groups, approaching levels observed in never-smokers, whereas participants who continued to smoke CCs exhibited stable or unfavorable changes throughout the study. Overall, the authors conclude that sustained substitution of smoking with a HTP consistently reduces exposure to smoke toxicants and is associated with improvements in multiple early biological indicators of risk, suggesting a reasonable likelihood of reduced tobacco-related disease risk compared with continued CC use, although without equating to the full benefits of smoking cessation [16].

Reduced Exposure Claims, Consumer Perceptions, and the Role of HTPs in Smoking Substitution or Cessation

Table 3 summarizes studies evaluating reduced exposure claims, consumer perceptions, and the role of HTPs in smoking substitution or cessation.

**Table 3:** Summary of studies evaluating reduced exposure claims, consumer perceptions, and the role of HTPs in smoking substitution or cessation, with the corresponding SORT classification. HTP: Heated tobacco product; CC: Conventional cigarette; SORT: Strength of Recommendation Taxonomy.

Study	Result	SORT
Cheng et al. 2025 [7] (industry-sponsored study)	(1) Public perception, largely shaped by the tobacco industry, is that HTPs are “less harmful,” reflecting a reduced – though not absent – risk perception. This perception may facilitate partial or complete switching from CCs to HTPs. (2) The role of HTPs in smoking cessation remains uncertain: while they may promote substitution of CCs and reduce consumption among dual users, particularly in smokers who are resistant to cessation therapies, the risk of prolonged dual use raises concerns regarding their true impact on sustained smoking cessation.	Strength C, Level 2
Audrain-McGovern et al. 2025 [25] (independent study)		Strength B, Level 1
DeAtley et al. 2025 [8] (independent study)		Strength B, Level 1
Suzuki et al. 2023 [6] (independent study)		Strength A, Level 1

Cheng et al. conducted a cross-sectional survey of 688 adults assessing the history of use of multiple tobacco products (including HTPs), risk perceptions, and understanding of modified risk messages (MRMs). Among 329 former smokers, 87.8% had smoked within the 30 days preceding their first use of a HTP, suggesting that smoking cessation occurred after HTP initiation. HTPs were perceived as less harmful than CCs (mean risk scores of 43.8 vs. 64.4, respectively). Understanding of MRMs was high, with 80.7% of participants correctly recognizing that complete switching reduces exposure to harmful chemicals, and 84.9% of these also identifying the need for full cessation of CC use. These findings indicate that HTPs may facilitate the transition of adult smokers - including those who did not use or did not succeed with conventional cessation treatments, by offering a potentially less harmful alternative accompanied by adequate understanding of its risk profile [7].

Audrain-McGovern et al.* *conducted a 21-day longitudinal study involving 87 smokers aged 18 to 65 years, evaluating the impact of complete substitution of CCs with HTPs over a 14-day period. The results demonstrated a significant increase in motivation for smoking cessation, suggesting that this may emerge as a direct consequence of HTP use, particularly when accompanied by a perception of reduced risk and substantial replacement of CC consumption. However, among heavier smokers, the degree of substitution was lower, which attenuated the effects on motivation, indicating that higher nicotine dependence limits HTP uptake and reduces their effectiveness as a substitute for this subgroup of smokers [25].

In a similar study with a smaller sample size (n = 27), DeAtley et al.* *observed a significant reduction in daily CC consumption and a 22.8% increase in motivation for smoking cessation. Perceived lower risk associated with HTPs did not directly explain this reduction but contributed indirectly by encouraging product uptake. However, the majority of participants did not achieve complete switching, reflecting high rates of dual use (96%) [8].

In a systematic review of 134 studies on HTPs, Suzuki et al. concluded that the current evidence in the biomedical literature is strongly influenced by the tobacco industry. Claims that HTPs are “safer” or more “desirable” than CCs are not supported by robust clinical morbidity and mortality data, but rather rely primarily on outcomes based on biomarkers and chemical exposure. The authors emphasize the need for further independent studies focused on real clinical endpoints to enable an objective assessment of the risks associated with HTP use [6].

Discussion

Based on the currently available evidence regarding the risk of LC in users of HTPs compared with CC smokers, studies suggest a substantial reduction in the formation of and exposure to carcinogenic compounds associated with HTP use. Chemical analyses indicate that these devices generate less than half of the carcinogenic compounds identified in CC smoke, with particularly notable reductions observed for acrylamide, whose emissions may be approximately fivefold lower. This reduced toxic burden translates, within available models, into significantly lower cancer risk estimates, with relative values ranging from approximately 3% to 4% of the risk estimated for CC smokers. However, it is important to emphasize that these estimates are derived predominantly from toxicological models and exposure-based risk assessments, and that robust evidence from long-term clinical or epidemiological studies confirming an actual reduction in LC incidence associated with HTP use is still lacking.

With respect to biomarker-based evidence, available studies demonstrate statistically significant reductions in several BoE and BoPH among HTP users compared with CC smokers. These include COHb, urinary NNAL, WBC count, and 8-epi-PGF2α. Such reductions suggest lower systemic and inflammatory toxicity in HTP users relative to CC smokers. However, although the direction of these changes is similar to that observed following smoking cessation, biomarker levels consistently remain higher than those recorded in never-smokers, indicating the persistence of residual risk.

Regarding consumer perceptions and the potential role of HTPs in smoking cessation or substitution, the literature indicates that these products are generally perceived as less harmful than CCs, reflecting a reduced risk perception. This perception may facilitate partial or complete switching from CCs to HTPs. However, the impact of HTPs on smoking cessation remains uncertain. While some studies suggest reductions in CC consumption, a substantial risk of prolonged dual use, characterized by concurrent use of HTPs and CCs, has been observed, which may undermine sustained cessation. Consequently, although HTPs are often regarded as reduced-risk products, this perception may paradoxically contribute to the continued use of nicotine and discourage complete smoking cessation.

This review has limitations inherent to its design as an evidence-based narrative review. Although a structured search strategy and predefined criteria were applied, the absence of a fully systematic methodology (e.g., PRISMA adherence and protocol registration) may limit reproducibility. The search strategy relied exclusively on the MeSH term “Heated Tobacco”, which was selected to maximize sensitivity; however, studies not yet indexed under this term or using alternative terminology may not have been captured. Additionally, the available evidence is predominantly based on surrogate biomarkers rather than long-term clinical outcomes. Of the 26 included studies, 12 (46.2%) were classified as industry-sponsored or industry-linked based on declared funding or author affiliations, which may introduce potential funding-related bias. Finally, the relatively recent introduction of HTPs limits the availability of long-term epidemiological data on LC incidence.

The findings of this review have important implications for clinical practice and public health. Healthcare professionals should therefore exercise caution when advising patients, and regulatory bodies should avoid framing these products as safe alternatives, particularly given the high prevalence of dual use. Physicians should also systematically inquire about HTP use when screening for tobacco consumption and provide evidence-based counselling regarding their safety profile and residual risks.

## Conclusions

HTPs are associated with reduced exposure to carcinogenic compounds and favorable changes in several biomarkers compared with CCs, suggesting the possibility of a lower LC risk. However, current estimates of reduced LC risk rely primarily on toxicological models and surrogate biomarkers rather than long-term clinical or epidemiological evidence and therefore remain uncertain. Although HTPs may contribute to partial harm reduction among smokers who completely switch, they are not risk-free and are frequently associated with dual use, which may limit potential health benefits. Robust independent longitudinal studies are essential to determine their true impact on LC incidence and broader public health outcomes.

## Appendices

Strength of Recommendation Taxonomy (SORT)

**Table 4:** Strength of recommendation grades * — Patient-oriented evidence measures outcomes that matter to patients: morbidity, mortality, symptom improvement, cost reduction, and quality of life. Disease-oriented evidence measures intermediate, physiologic, or surrogate endpoints that may or may not reflect improvements in patient outcomes (e.g., blood pressure, blood chemistry, physiologic function, and pathologic findings).

Strength of recommendation	Definition
A	Recommendation based on consistent and good-quality patient-oriented evidence*.
B	Recommendation based on inconsistent or limited-quality patient-oriented evidence*.
C	Recommendation based on consensus, usual practice, opinion, disease-oriented evidence,* and case series for studies of diagnosis, treatment, prevention, or screening.

**Table 5:** Assessing quality of evidence SR: Systematic Review; RCT: Randomized Controlled Trial. ** — High-quality diagnostic cohort study: cohort design, adequate size, adequate spectrum of patients, blinding, and a consistent, well-defined reference standard. † — High-quality RCT: allocation concealed, blinding if possible, intention-to-treat analysis, adequate statistical power, adequate follow-up (greater than 80 percent). †† — An all-or-none study is one where the treatment causes a dramatic change in outcomes, such as antibiotics for meningitis or surgery for appendicitis, which precludes study in a controlled trial.

Study quality	Diagnosis	Treatment/prevention/screening	Prognosis
Level 1: good-quality patient-oriented evidence	Validated clinical decision rule; Systematic Review (SR)/meta-analysis of high-quality studies; High-quality diagnostic cohort study**.	SR/meta-analysis or Randomized Controlled Trial (RCT) with consistent findings; High-quality individual RCT†; All-or-none study††.	SR/meta-analysis of good-quality cohort studies; Prospective cohort study with good follow-up.
Level 2: limited-quality patient-oriented evidence	Unvalidated clinical decision rule; SR/meta-analysis of lower quality studies or studies with inconsistent findings; Lower quality diagnostic cohort study or diagnostic case-control study**.	SR/meta-analysis of lower quality clinical trials or of studies with inconsistent findings; Lower quality clinical trial†; Cohort study; Case-control study.	SR/meta-analysis of lower quality cohort studies or with inconsistent results; Retrospective cohort study or prospective cohort study with poor follow-up; Case-control study; Case series.
Level 3: other evidence	Consensus guidelines, extrapolations from bench research, usual practice, opinion, disease-oriented evidence (intermediate or physiologic outcomes only), and case series for studies of diagnosis, treatment, prevention, or screening.		

## References

[1] Braznell S, Campbell J, Gilmore AB (2024). What can current biomarker data tell us about the risks of lung cancer posed by heated tobacco products?. Nicotine Tob Res.

[2] Lee T, Park J, Kim Y, Chen WH, Kwon EE (2025). Comparison of toxic pyrogenic compounds derived from conventional cigarettes and heated tobacco products. J Hazard Mater.

[3] Dusautoir R, Zarcone G, Verriele M (2021). Comparison of the chemical composition of aerosols from heated tobacco products, electronic cigarettes and tobacco cigarettes and their toxic impacts on the human bronchial epithelial BEAS-2B cells. J Hazard Mater.

[4] Śniadach J, Kicman A, Michalska-Falkowska A, Jończyk K, Waszkiewicz N (2025). Changes in concentration of selected biomarkers of exposure in users of classic cigarettes, e-cigarettes, and heated tobacco products—a narrative review. Int J Mol Sci.

[5] Otsuka Y, Kaneita Y, Itani O, Matsumoto Y, Hatori Y, Imamura S (2023). Awareness, attitudes, and concerns regarding heated tobacco products among physicians in Japan. J Epidemiol.

[6] Suzuki H, Aono N, Zhang Y (2024). Comparison of publications on heated tobacco products with conventional cigarettes and implied desirability of the products according to tobacco industry affiliation: a systematic review. Nicotine Tob Res.

[7] Cheng HG, Noggle B, Vansickel AR, Largo EG, Magnani P (2025). Tobacco use, risk perceptions, and characteristics of adults who used a heated tobacco product (IQOS) in the United States: cross-sectional survey study. JMIR Form Res.

[8] DeAtley T, Stone MD, Strasser AA, Audrain-McGovern J (2024). The role of IQOS risk perceptions on cigarette smoking behaviours: results from a prospective pilot study. Tob Control.

[9] Znyk M, Jurewicz J, Kaleta D (2021). Exposure to heated tobacco products and adverse health effects: a systematic review. Int J Environ Res Public Health.

[10] Rodrigo G, Jaccard G, Tafin Djoko D, Korneliou A, Esposito M, Belushkin M (2021). Cancer potencies and margin of exposure used for comparative risk assessment of heated tobacco products and electronic cigarettes aerosols with cigarette smoke. Arch Toxicol.

[11] Sato A, Ishigami A (2023). Effects of heated tobacco product aerosol extracts on DNA methylation and gene transcription in lung epithelial cells. Toxicol Appl Pharmacol.

[12] Esposito F, Squillante J, Nolasco A, Montuori P, Macrì PG, Cirillo T (2022). Acrylamide levels in smoke from conventional cigarettes and heated tobacco products and exposure assessment in habitual smokers. Environ Res.

[13] Lee PN, Coombs KJ, Fry JS (2025). Estimating lung cancer risk from e-cigarettes and heated tobacco products: applications of a tool based on biomarkers of exposure and of potential harm. Harm Reduct J.

[14] Ansari SM, Leroy P, de La Bourdonnaye G, Pouly S, Reese L, Haziza C (2025). Differences in biomarkers of potential harm after 2+ years of tobacco heating system use compared to cigarette smoking: a cross-sectional study. Biomarkers.

[15] Ansari SM, Hession PS, David M, Blanc N, de La Bourdonnaye G, Pouly S, Haziza C (2024). Impact of switching from cigarette smoking to tobacco heating system use on biomarkers of potential harm in a randomized trial. Biomarkers.

[16] Gale N, McEwan M, Hardie G, Proctor CJ, Murphy J (2022). Changes in biomarkers of exposure and biomarkers of potential harm after 360 days in smokers who either continue to smoke, switch to a tobacco heating product or quit smoking. Intern Emerg Med.

[17] Gale N, McEwan M, Camacho OM, Hardie G, Proctor CJ, Murphy J (2021). Changes in biomarkers after 180 days of tobacco heating product use: a randomised trial. Intern Emerg Med.

[18] Gale N, McEwan M, Camacho OM, Hardie G, Murphy J, Proctor CJ (2021). Changes in biomarkers of exposure on switching from a conventional cigarette to the glo tobacco heating product: a randomized, controlled ambulatory study. Nicotine Tob Res.

[19] Sakaguchi C, Nagata Y, Kikuchi A, Takeshige Y, Minami N (2021). Differences in levels of biomarkers of potential harm among users of a heat-not-burn tobacco product, cigarette smokers, and never-smokers in Japan: a post-marketing observational study. Nicotine Tob Res.

[20] Camacho OM, Hedge A, Lowe F, Newland N, Gale N, McEwan M, Proctor C (2020). Statistical analysis plan for "a randomised, controlled study to evaluate the effects of switching from cigarette smoking to using a tobacco heating product on health effect indicators in healthy subjects". Contemp Clin Trials Commun.

[21] Bosilkovska M, Tran CT, de La Bourdonnaye G, Taranu B, Benzimra M, Haziza C (2020). Exposure to harmful and potentially harmful constituents decreased in smokers switching to carbon-heated tobacco product. Toxicol Lett.

[22] Yuki D, Kikuchi A, Suzuki T, Sakaguchi C, Huangfu D, Nagata Y, Kakehi A (2022). Assessment of the exposure to selected smoke constituents in adult smokers using in-market heated tobacco products: a randomized, controlled study. Sci Rep.

[23] McKelvey K, Baiocchi M, Halpern-Felsher B (2020). PMI's heated tobacco products marketing claims of reduced risk and reduced exposure may entice youth to try and continue using these products. Tob Control.

[24] Chen-Sankey JC, Kechter A, Barrington-Trimis J (2023). Effect of a hypothetical modified risk tobacco product claim on heated tobacco product use intention and perceptions in young adults. Tob Control.

[25] Audrain-McGovern J, Klapec O, Paul Wileyto E, Strasser AA (2025). Shifts in motivation to quit cigarette smoking associated with IQOS use. Addict Behav.

[26] Tompkins CN, Burnley A, McNeill A, Hitchman SC (2021). Factors that influence smokers' and ex-smokers' use of IQOS: a qualitative study of IQOS users and ex-users in the UK. Tob Control.

[27] Ebell MH, Siwek J, Weiss BD (2004). Strength of recommendation taxonomy (SORT): a patient-centered approach to grading evidence in the medical literature. Am Fam Physician.

